# O10.5. ABNORMAL MODULAR ORGANIZATION OF THE FUNCTIONAL CONNECTOME PREDICTS CONVERSION TO PSYCHOSIS IN CLINICAL HIGH-RISK YOUTH

**DOI:** 10.1093/schbul/sby015.257

**Published:** 2018-04-01

**Authors:** Guusje Collin, Larry Seidman, Matcheri S Keshavan, Zhenghan Qi, William S Stone, TianHong Zhang, Yingying Tang, Martha E Shenton, Jijun Wang, Susan Whitfield-Gabrieli

**Affiliations:** 1 University Medical Center Utrecht; 2 Harvard Medical School; 3 Massachusetts Mental Health Center, Beth Israel Deaconess Medical Center, Harvard Medical School; 4 University of Delaware; 5 Harvard Medical School, Beth Israel Deaconess Medical Center; 6 Shanghai Mental Health Center, Shanghai Jiao Tong University School of Medicine; 7 Brigham and Women’s Hospital, Harvard Medical School, Veterans Affairs Boston Healthcare System; 8 Massachusetts Institute of Technology, McGovern Institute for Brain Research

## Abstract

**Background:**

The first episode of schizophrenia is typically preceded by a prodromal phase characterized by sub-threshold symptoms and declining functioning. Elucidating the neurobiological substrate of prodromal symptoms that progress into overt psychotic illness is crucial to the development of early detection and intervention strategies for schizophrenia. In this study, we performed a functional connectome analysis in a large group of adolescents and young adults at Clinical High Risk (CHR) for schizophrenia. We aim to assess whether, and if so how, baseline connectome organization distinguishes CHR youth that go on to develop psychosis.

**Methods:**

This study comprises a total of 251 subjects, including 158 psychotropically-naïve CHR subjects (CHRs) and 93 healthy controls (HCs), who were matched to CHRs on age, gender, and level of education. Prodromal symptoms and cognition were assessed using the SIPS structured interview and MATRICS cognitive battery. Anatomical T1 MRI and resting-state fMRI scans were collected at baseline and processed using Freesurfer v6.0 and CONN v17.d software. For each subject, a functional connectome map was reconstructed consisting of 162 nodes representing 148 cortical regions from the Destrieux atlas and 14 subcortical structures. Functional connectomes were analyzed in terms of modular topology using the Louvain community detection method. Modular network partitions of individual CHRs were compared to a group-averaged HC network using the rand similarity coefficient (SR), providing a measure of the level of (ab)normality of the CHRs’ modular partitions. Analysis of covariance (correcting for age- and gender) was used to compare SR levels between CHRs who developed psychosis during follow-up (CHR+; N = 23) as compared to CHRs who did not develop psychosis (CHR-; N = 135). Kaplan-Meier analysis was used to estimate psychosis-free survival functions for CHRs with below- versus above-average SR, which were compared using log-rank tests. Cox regression analysis was used to assess how baseline connectome organization and clinical measures (i.e., demographics, symptoms, IQ) predicted time to conversion.

**Results:**

Modular community detection in HCs yielded five major modules including a posterior ‘visual’, central ‘sensorimotor’, medial frontoparietal ‘default-mode’, lateral frontoparietal ‘central-executive’, and inferior ‘limbic’ module. Modular connectome organization of CHR+ was significantly less similar to HCs than CHR- (F(1,154) = 7.14, p = 0.008). A region-specific analysis to identify which regions contributed most to aberrant modular connectome organization in CHR+ showed that superior temporal (including STG), medial temporal (including amygdala), and ventromedial prefrontal regions were most abnormal in terms of their modular assignment. Psychosis-free survival functions of CHRs with low versus high SR were significantly different (z = 2.5, p = 0.013), with a Hazard ratio of 3.3 indicating an over 3-fold relative event rate (i.e., conversion to psychosis) in CHRs with abnormal baseline connectome organization. Cox regression analysis indicated that baseline connectome organization (z = -2.3, p = 0.019), IQ (z = -2.7, p = 0.007), and gender (z = 2.0, p = 0.048) predicted time to conversion.

**Discussion:**

This study indicates that abnormalities in functional connectome organization precede the first psychotic episode. Conversion to psychosis was found to be over three times more likely in CHRs with abnormal modular organization of the functional connectome at baseline. Our results suggest that functional connectome reorganization may underlie the gradual manifestation of prodromal symptoms. These findings may contribute to early diagnosis and intervention in schizophrenia.

